# Recent advances in biomimetic nanodelivery systems for cancer Immunotherapy

**DOI:** 10.1016/j.mtbio.2025.101726

**Published:** 2025-04-05

**Authors:** Jiawei Yang, Xueqi Li, Tongyu Li, Jin Mei, Ying Chen

**Affiliations:** a Cixi Biomedical Research Institute, Wenzhou Medical University, Zhejiang, China, No. 508 North Second Ring East Road, Ningbo, 315302, Zhejiang, China; b Department of Hematology, The First Affiliated Hospital of Ningbo University, 59 Liuting Street, Haishu District, Ningbo, 315010, China; c Institute of Engineering Medicine, The First Affiliated Hospital of Ningbo University, 59 Liuting Street, Haishu District, Ningbo, 315010, China

**Keywords:** Biomimetic delivery system, Nanodrug, Tumor immune microenvironment, Immunotherapy, Engineering modification

## Abstract

Tumor immunotherapy is a developing and promising therapeutic method. However, the mechanism of tumor immune microenvironment and individual differences of patients make the clinical application of immunotherapy still very limited. The resulting targeting of the tumor environment and immune system is a suitable strategy for tumor therapy. Biomimetic nanodelivery systems (BNDS) coated with nanoparticles has brought new hope for tumor immunotherapy. Due to its high targeting, maximum drug delivery efficiency and immune escape, BNDS has become one of the options for tumor immunotherapy in the future. BNDS combines the advantages of natural cell membranes and nanoparticles and has good targeting properties. This review summarizes the relationship between tumor and immune microenvironment, classification of immunotherapy, engineering modification of cell membrane, and a comprehensive overview of different types of membrane BNDS in immunotherapy. Furthermore, the prospects and challenges of biomimetic nanoparticles coated with membranes in tumor immunotherapy are further discussed.

## Graphical abstract

To further enhance the efficacy of cancer immunotherapy, biomimetic nanodelivery systems (BNDS) has attracted widespread attention. This review summarizes the advancements in tumor immunotherapy and the application of various membrane-coated or engineered-modified BNDS in immunotherapy. Moreover, the preparation and characterization of BNDS are also elucidated.

**Figure:**
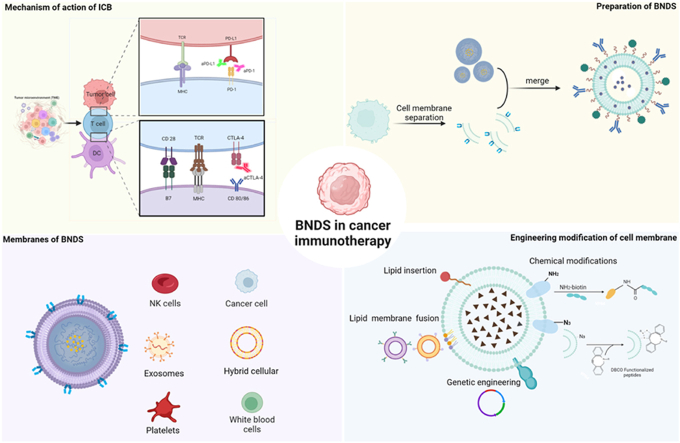

## 1 Introduction

The incidence and mortality rates of malignant tumors are rising annually, making them the second leading cause of death globally. Despite numerous clinical methodologies for cancer treatment emerging in recent years, the intricate physiological and pathological mechanisms of cancer, influenced by multiple factors, continue to pose significant challenges, the recurrence and metastasis of tumor is still a big problem [[1], [2]]. Clinical treatment modalities include surgery, radiotherapy, palliative care, and emerging therapeutic approaches such as cell therapy, cancer vaccines and gene therapy. These treatment modalities can improve the survival rate of patients to some extent, but they are often accompanied by various adverse reactions and recurrence resistance problems [3]. For instance, surgical resection is only applicable to early-stage tumors, radiotherapy may damage the adjacent normal tissues through ionizing radiation; patients with recurrence after chemotherapy often face rapid disease progression [4]. Therefore, there is still considerable progress needed to develop new treatments to improve anti-tumor efficacy.

In the field of medical research, tumor immunotherapy is considered to be a promising cancer treatment method, playing the role of "police" within the human body, using the body's own immune system to identify and destroy cancer cells. With recent advancements in our understanding of immune system mechanisms and medical technology, immunotherapy has emerged as a pivotal means of treating malignant tumors [5]. Immunotherapy can also be combined with traditional methods, such as radiation therapy, chemotherapy and other means to develop personalized treatment plans suited to individual patients. Tumor immunotherapy has been widely recognized, with the continuous development of effective immune agents, it holds the potential to become the most promising avenue for cancer treatment. The advantages of immunotherapy are: a. compared with traditional drug therapy, the side effects are relatively small [6]; b. Immunotherapy can treat a wide range of cancer categories, including breast cancer, cervical cancer, lung cancer, gastric cancer, HPV, liver metastasis and other cancers can be applied [7]; c. Immunotherapy has a long duration of effect [8]; d. Effective treatment is available at different stages of cancer patients, including early, middle and late-stage patients [9]. In the tumor microenvironment, immune cells interact with tumor cells to regulate cancer growth and facilitate immune escape. For the first time, the Food and Drug Administration (FDA) has approved various therapeutic strategies, including immune checkpoint blockade (ICB) therapy, adoptive cell therapy (ACT), and tumor vaccines, which have been successfully implemented in clinical practice [10]. Although immunotherapy has significant advantages, it also has some limitations, such as the possibility of overactive immune response due to immune checkpoint blocking [11], the high time-consuming preparation of tumor vaccines [[12], [13]]. Therefore, these issues need to be addressed urgently.

Nanoparticles (NPs) play an important role in the field of diagnosis and treatment, and also introduce novel methodologies for cancer therapeutic immunity and immunotherapy. NPs can increase the stability, circulation time and safety of drugs within the body [14]. Typically, the particle size of nanoparticles is between 1 and 1000 nm, which has good drug delivery efficiency. Compared with traditional chemotherapy drugs, duing to their minute particle size, extensive surface area, low toxicity, and brief half-life, NPs have been extensively utilized in the treatment of diverse diseases [15]. In recent years, nanoparticle drug delivery system has shown great potential in the process of cancer treatment. BNDS inspired by nature, the cell membrane is coated on NPs, including red blood cells, tumor cells, macrophages, neutrophils and so on [16]. The combination of nanoparticle and cell membrane can achieve a "camouflage state" to avoid the host immune response [[17], [18], [19], [20]]. It finds applications in diagnostic imaging, chemotherapy, immunotherapy, and other avenues. In this comprehensive review, the interaction between tumor immune microenvironment and immunotherapy, the types of immunotherapy, the preparation and characterization of BNDS, and the application of BNDS in tumor immunotherapy are reviewed. Lastly, the prospects and challenges associated with BNDS are explored.

## 2 Immunotherapy of tumors

The fundamental principle of tumor immunotherapy is to activate and enhance the patient's own immune system to recognize and eliminate cancer cells. Although immunotherapy has shown good efficacy in some types of cancer, its effectiveness remains limited in many patients, especially in those with complex tumor microenvironments and immunosuppressive tumors [21]. Therefore, in-depth research on the current status and future development directions of tumor immunotherapy is particularly important. Currently, the main types of tumor immunotherapy include non-specific immune stimulation therapy, immune checkpoint inhibitors, cell therapy, and tumor vaccines, etc [22].

### 2.1 Tumor immune microenvironment

In the tumor immune microenvironment, immune cells constitute a critical component. Key immune cell types include T cells, B cells, tumor-associated macrophages (TAMs), natural killer (NK) cells, and dendritic cells. TAMs exhibit a dual role in the tumor microenvironment, capable of both promoting tumor growth and activating anti-tumor immune responses [23]. Research indicates that the polarization state of TAMs (M1 and M2 phenotypes) significantly influences tumor progression. Specifically, M1-type macrophages exert anti-tumor effects, whereas M2-type macrophages facilitate tumor growth and metastasis [24]. T cells play a pivotal role in the tumor microenvironment; CD8^+^ cytotoxic T cells are responsible for recognizing and eliminating tumor cells, while CD4^+^ helper T cells modulate the functions of other immune cells through cytokine secretion [25]. However, immune suppressive factors within the tumor microenvironment can impair T cell function, thereby diminishing the efficacy of anti-tumor immune responses [26]. Additionally, NK cells are crucial in tumor immunity, participating in anti-tumor immune responses by directly lysing tumor cells and secreting cytokines. Studies have shown that cytokines within the tumor microenvironment can influence NK cell activity and function, thus impacting tumor progression [27].

Intercellular interactions within the tumor microenvironment significantly influence the function of immunosuppressive cells. For example, tumor cells can modulate the polarization state of TAMs by secreting exosomes and cytokines, promoting their conversion to the M2 phenotype, thereby enhancing tumor immune evasion [28]. Tumor cells can also evade immune surveillance by altering the expression of tumor-associated antigens. Specifically, they downregulate the expression of major histocompatibility complex (MHC) molecules, reducing antigen presentation and evading recognition by T cells [29]. Additionally, tumor cells may suppress T cell activity by upregulating immune checkpoint molecules such as PD-L1, further facilitating immune escape [30]. In certain conditions, tumor cells can exploit immunosuppressive factors in the tumor microenvironment to alter their antigen expression. For instance, under hypoxic conditions, tumor cells may activate hypoxia-inducible factor (HIF-1α) to regulate antigen expression, thereby impacting the recognition capability of immune cells [31]. Therefore, targeting these immunosuppressive cells or the factors they secrete represents a promising strategy to enhance the therapeutic efficacy of cancer treatments.

### 2.2 Non-specific immunostimulation

Nonspecific immune stimulation refers to the activation of the immune system through certain substances or methods to enhance its overall immune response, rather than targeting specific antigens. This type of stimulation can broadly activate T cells and B cells, independent of the specificity of their receptors [32]. By stimulating T lymphocytes or antigen-presenting cells, the antigen-presenting process is augmented. This includes Lymphokine-Activated Killer (LAK) cell therapy and Cytokine-Induced Killer (CIK) cell therapy. LAK cell therapy involves the cultivation of peripheral blood mononuclear leukocytes in vitro, which are then expanded and activated by the lymphokine IL-2 before being reintroduced into the patient to enhance their tumoricidal effect [33]. CIK therapy is induced by a variety of methods. Compared with LAK therapy, the expansion rate exhibits a faster expansion rate and higher tumoricidal activity [34]. However, due to the non-specific nature of their tumoricidal action, these therapies cannot achieve complete tumor eradication, leading to their decreasing clinical application.

### 2.3 Immune checkpoint blocking

**Fig. 1:**
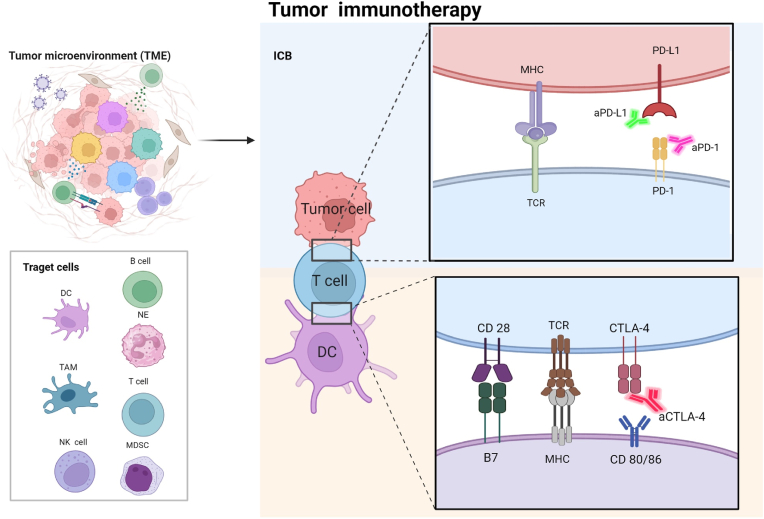
Target cells of immunotherapy and mechanism of action of ICB. The combination of PD-1 and PD-L1 inhibits the proliferation of T cells and the secretion of cytokines. The use of monoclonal antibodies against PD-1/PD-L1 (aPD-1/aPD-L1) can restore the anti-tumor activity of T cells. CTLA-4 is homologous to CD28 and can both bind to B7 molecules or CD60/86 on APCs, thereby inhibiting T cell activity. The use of CTLA-4 blockers (aCTLA-4) can restore T cell function. Created with BioRender.com.

Immune checkpoint blockade is a therapeutic strategy that restores or enhances the ability of immune cells, particularly T cells, to target and attack tumor cells by inhibiting suppressive signaling pathways within the immune system [35]. The activity of immune checkpoints is mainly regulated by the regulation of T cells, which recognize specific antigens through surface-related receptors, receive positive and negative signals of stimulation, thereby determining whether the cell becomes activated or suppressed. They participate in various stages of the T cell response [36]. Under normal physiological conditions, the activation and inhibition pathways of T cells maintain a delicate balance. However, tumors can evade the immune system by binding to their respective ligands and T cells, thereby inducing immune cell activity or mediating immune cell apoptosis [37]. Immune checkpoint blocking (ICB) exerts anti-tumor effects by overcoming tumor immune escape(Fig. 1). Three distinct immunoscreening inhibitors, namely CTLA-4, PD-1, and PD-L1 inhibitors, have been approved by the FDA for the treatment of various tumor types. Although the cancer blocking system administration of CTLA-4, PD-1/L1 is still significant, non-immunogenic and immunosuppressive tumor microenvironment remains severely hindered [38]. A number of other immune checkpoint inhibitors (ICIs), such as lymphocyte activation Gene-3 and T cell Ig-3, exist, but their usage is not widespread. Nevertheless, ICIs are non-specific and are often associated with adverse reactions [39]. Therefore, the precise delivery of ICIs to the tumor site is considered a promising approach to increase drug concentration at the target site and reduce the adverse reactions associated with ICIs [40].

CTLA-4/CD152 is the first clinically targeted immune checkpoint receptor, but its mechanism of action remains unclear. CTLA-4 competitively binds to the B7 molecule (CD80 and CD86) homologically with CD28, whereas CTLA-4 has a higher affinity, 20 times that of CD28. CTLA-4 transmits inhibitory signals to T cells and inhibits T cell activation, while CD28 transmits stimulatory signals and activates T cells. Therefore, inhibiting the binding of CTLA-4 and B7 can enhance the effect of immunotherapy [41]. In tumor cells, B7 is overexpressed, recognizing and binding to CTLA-4, downregulating the immune response of T cells, and leading to immune escape. CTLA-4 monoclonal antibody blocks the binding of CTLA-4 and B7 by binding to CTLA-4, promotes the binding of CD28 and B7, activates T cells, and enhances immune recognition and killing [42]. CTLA-4 monoclonal antibody (Nb16) has achieved remarkable results in the treatment of advanced melanoma. For example, CTLA-4 specific nano-antibody CTLA-4 NPs was screened from high-quality dromedary immune bank by phage-display technology, which enabled CTLA-4 to be rapidly upregulated and bound to B7 molecule with higher affinity than CD28. Delayed the growth of melanoma and prolonged the survival time of C57BL/6 mice with B16 melanoma [43]. There have been several cases involving monoclonal antibodies, with the antibody CTLA-4 Ipilimumab being approved by the FDA in 2011.

PD-1 (anti-programmed cell death-1), also referred to as CD279, belongs to the immunoglobulin B7-CD28 family and facilitates the expression of T and B cells through interaction with its ligands, PD-L1 and PD-L2. PD-L1 is also highly expressed in tumor cells, including lung cancer cells, colon cancer, etc [44]. When PD-1 binds to PD-L1, it inhibits the inactivation of the RAS/MEK/ERK signaling pathway and arrests the cell cycle [45]. Additionally, PD-L1 can induce IL-10-mediated immunosuppression by stimulating T cells, thereby suppressing their proliferation and activity, leading to T cell inactivation and exhaustion [46]. Conversely, blocking the PD-1/PD-L1 signaling pathway can reverse the tumor immune tolerance microenvironment, exert endogenous anti-tumor immune effect and inhibit tumor cells [47]. At present, more than a dozen monoclonal antibodies targeting PD-1 or PD-L1 have been marketed for the treatment of non-small cell lung cancer, rectal cancer, colon cancer and melanoma [48]. Nivolumab, which is also a monoclonal antibody to PD-1, has been shown to significantly improve survival in melanoma patients [49]. Triprilimab is a humanized IgG4 PD-1 monoclonal antibody, which has significant efficacy in the treatment of stage II/III NSCLC. The survival rate of patients who received induced ICI treatment before radical CRT and consolidated ICI treatment was significantly higher than that of patients [50]. Despite its effectiveness, this antibody has certain drawbacks, such as high cost and limited stability, as non-specific binding may lead to autoimmunity or other adverse reactions. There is a pressing need to harness nanotechnology to enhance its efficacy in cancer treatment and mitigate systemic adverse reactions [51].

### 2.4 Cellular immunotherapy

Adoptive cell transfer therapy is a cancer treatment in which immune cells are engineered in vitro, amplified and then transfused back into the patient to stimulate an effective immune response and kill tumors. T cell populations are the main candidates for this kind of precision cell immunotherapy, such as tumor infiltrating lymphocyte, chimeric antigen receptor T cell, CAR⁃T), T cell receptor⁃gene engineered T cell (TCR⁃T), etc. CAR-T therapy, a subtype of adoptive T cell therapy, is currently one of the most promising therapies for hematologic malignancies [[52], [53]].

Between 1989 and 1993, Israeli immunologists Zelig Eshhar and Gideon Gross developed CAR-T, which developed the first clinically useful generation of CAR-T cells. Subsequently, the antitumor activity of CAR-T cells was improved, and the effector function and in vivo persistence of the third generation were improved. The anti-tumor efficacy, ligands and enzymes capable of degrading solid tumors were further enhanced in the fourth generation of CAR-T [54]. In 2017, Novartis' tisagenlecleucel was approved by the FAD for the treatment of B-cell lymphoma in children and young adults. And in 2020, Gilead's brexucabtagene autoleucel was approved for the treatment of adult B-cell lymphoma [55]. Producing CAR-T cells requires several steps. Initially, white blood cells are extracted, followed by the collection and separation of T cells from these white blood cells. Subsequently, the subsets of T cells at the CD4/CD8 level are isolated, each step being of paramount importance.

CLL-1 (C-type lectin-like molecule 1) is a protein selectively present on leukemia stem cells in acute myeloid leukemia (AML) and absent in normal hematopoietic stem cells, making it an exceptional therapeutic target [56]. To address AML, Wang et al. expressed the type II transmembrane glycoprotein CLL-1 in leukemia stem cells (LSC). In animal models, mice treated with CLL-1 CAR T cells showed a significant reduction in systemic leukemia burden and a significant reduction in tumor cells compared to controls [57]. CD126 is a marker of many malignant tumors. 293T cells were packaged with lentivirus and transfected with human T cells. The resulting CD126 CAR T cells killed many tumor cells in an antigen-specific manner. Binding with sIL-6R can alleviate cytokine release syndrome and keep tumor growth under control [58]. Furthermore, there are approaches that combine CAR-T therapy with radiation therapy. Patients with triple-negative breast cancer have higher express EGFR receptors. The combination of EGFR CAR-T cell therapy and radiation therapy leads to a significant increase in the number of tumor-infiltrating CAR-T cells without elevating the risk of cytokine release syndrome, thereby promoting CAR-T cell infiltration and lethality [59]. Despite the clinical application of CAR-T cells, there remain numerous limitations, including the potential for significant toxicity posing a serious risk to patients, immune escape, inadequate anti-tumor action, the optimal carrier for CAR-T cells, and long-term safety concerns [60].

### 2.5 Tumor vaccine

Tumor vaccines activate T cells in the patient's body by introducing an antigen, which in turn induces an immune response and kills the tumor [61]. Once injected into the body, the vaccine is recognized and captured by antigen-presenting cells (APCs), which then migrate to draining lymph nodes to induce the proliferation and differentiation of T lymphocytes and B lymphocytes. Finally, cytotoxic T lymphocytes (CTL) are secreted by antibodies to kill and eliminate antigen-targeting cancer cells [62]. Tumor vaccines are divided into preventive vaccines and therapeutic vaccines. According to the mechanism of action of the vaccine, it is further divided into whole cell vaccine, tumor polypeptide vaccine, gene engineering vaccine and antibody tumor vaccine [63]. Vaccines have proven effective in preventing human diseases, yet the traditional vaccine preparation process is relatively complex, potentially compromising vaccine immunogenicity and posing safety risks. In recent years, advancements in vaccine development have been made to combat the spread of malaria, AIDS, and other diseases. Notably, nanotechnology has been incorporated into the construction and application of new vaccines. Nanocarriers for vaccine delivery can not only improve the stability and immunogenicity of antigens, but also improve the efficiency of antigen delivery, which has become a common carrier [64].

In conclusion, among the numerous tumor treatment methods, immunotherapy represents an innovative and promising approach in clinical cancer therapy. The immune system and tumor cells engage in complex bidirectional interactions. While the immune system can induce tumor cell death, tumor cells can also evade immune recognition and attack through various adaptive mechanisms. However, with the deepening understanding of the intricate tumor immune microenvironment and the rapid advancement of BNDS, the biocompatibility and targeting capabilities of BNDS, particularly their ability to block immune checkpoints, ensure that tumor cells remain under continuous immune surveillance. Consequently, BNDS holds significant potential for enhancing tumor immunotherapy.

## 3 Preparation and characterization of biomimetic membrane

The separation of cell membranes depends on the type of treatment for different diseases, but the preparation of biomimetic nanosystems is summarized as three parts: a. The separation of the cell membrane b. The preparation of nanoparticles c. The assembly of the cell membrane coated with nanoparticles [65]. Biomimetic nanosystems replicate the biological functionalities of natural cells. The selection of biomimetic nanosystems originates from the distinctive properties of the unique components of the cell membrane and the distinctive proteins on its surface. The biomimetic strategy is particularly prominent [66]. The addressing of the blood-brain barrier for central nervous system diseases constitutes a significant challenge, and the high biocompatibility of nanomedicine offers a promising recourse for effective treatment strategies [67].

BNDS has many characteristics: a. good targeting and low adverse reactions. The surface composition of these nanosystems resembles that of the host cell membrane, enabling them to evade immune recognition, exhibit low immunogenicity, and reduce rapid clearance by the immune system. Consequently, they effectively minimize distribution in normal tissues while utilizing membrane surface proteins and molecules for targeted drug delivery. [68,69]. b. High dispersion. With a nanoparticle size of approximately 100 nm, their small particle size and surface structure constitute the advantages of nanomedicine. They can be administered intravenously and subsequently reach the lesion site through pinocytosis. The surface of nanoparticles is usually not saturated, and the existence of lone pair electrons makes them have high surface activity, so hydrophobic drugs can be embedded and the solubility of drugs can be improved [70]. c. Versatility. BNDS can carry a variety of types of therapeutic agents, while showing a variety of functions, the choice of cell membrane and its surface modification have significant advantages in functional imitation and multifunctional therapeutic delivery, such as magnetic nanoparticles, folic acid modification and soon [[71], [72]].

### 3.1 Separation of cell membrane

The membrane is a phospholipid bilayer structure, and the separation of the membrane is the process of separating the membrane and the contents in the membrane. In order to better realize the biomimetic nanoparticles in vivo targeting, circulation and other functions, the protein on the membrane should be preserved as much as possible to avoid the loss of the biological function of the membrane caused by protein denaturation. Now more commonly used: ultrasonic method, freeze-thaw method, extrusion method cracking method.

The ultrasonic method involves the use of ultrasound to broken cells. Typically, the ultrasonic frequency is controlled within the range of 20–50 KHz. Depending on the frequency of ultrasound, the degree of cell rupture varies [73]. This method is simple and the operation time is short. However, the advantages and disadvantages are also particularly obvious. The ultrasound process releases a lot of heat, which will lead to high temperature degeneration of the cell membrane. Therefore, an ice bath is necessary during the ultrasonic process. According to the different sources of cells, the regulation of different ultrasonic power, duration, and the interval between the ultrasonic switch need special consideration [74].

Freeze-thaw method is to make the cell in freezing (dry ice or ethanol bath) and thawing repeated process. During these cycles, the cells are destroyed. In the freezing process, ice crystals form and expand within the cells, while during thawing at room temperature, they undergo cold shrinkage. This results in rupture of the cell membrane and separation of its contents. This method is often combined with others [75]. This method is easy to harvest the membrane, but the membrane breaking is not complete, and the rate of membrane harvesting is low. In the process of repeated freezing, continuous low temperature and thawing, the proteins on the cell membrane will be damaged. Therefore, inhibitors can be added to prevent protein destruction during this process.

Extrusion method is to pass the cell suspension through the filter membrane with the required aperture, and break the cells into a large number of cell vesicles by physical extrusion. The pore size of the filter membrane, the pressure applied, and the number of extrusions will all affect the membrane harvest rate [76]. This method can retain the protein on the cell membrane to a large extent, but it has a large sample loss and is difficult to operate when the sample quantity is large, making it more suitable for preparing small samples.

Hypotonic lysis method is to use the cell swelling to rupture under low osmotic pressure, and then the method of separating the cell membrane. The main components of cracking buffer are buffer salt and ionic salt, which are used to adjust the pH and protect the enzymes and proteins on the cell membrane from cracking. Some also use 0.25 % PBS or 0.4 % NaCl as the lysate [77]. There are different classes of lysates depending on the type of cell. hypotonic lysis method is a relatively mild and ideal cleavage method, simple operation, short time consumption, and high efficiency of membrane acquisition, but it is difficult for large-scale cell extraction.

In practical applications, according to different cell types, the above methods are mixed, and through continuous control conditions, the most efficient way of cell membrane extraction is selected without destructing the protein on the cell membrane. Then through repeated washing and centrifugation to obtain high purity cell membrane.

### 3.2 Methods of membrane coating nanoparticles (Table 1)

Biomimetic nanoparticles can be synthesized through the coating of membranes with nanoparticles. During the coating process, the particle size, uniformity, membrane coverage and protein loss of biomimetic nanoparticles are all important factors to be investigated [78]. Thus, the biomimetic nanoparticles can play the functions of targeting and long circulation more effectively, enabling the bionic nanoparticles to reach the lesion site more. At present, many fusion methods have been proposed, and different coating technologies have their own characteristics, including extrusion method, ultrasonic method, microfluidic electroporation and so on [79].

Co-extrusion is a biomimetic nanosystem in which the purified membrane and nanoparticle mixture are repeated many times through polycarbonate porous membranes of different sizes, and the core-shell structure is obtained by repeated mechanical extrusion. It can not only be used to prepare a single type of cell membrane bionic nanoparticles, but also suitable for bionic nanosystems with hybrid cell membranes [80]. The method has the advantages of simple operation, high cell membrane coverage and uniform particle size, but an excessive amount of the cell membrane is lost during the operation. So it can only be applied to small batch production.

Ultrasound involves dispersing a mixture of cell membranes and nanoparticles under ultrasonic waves and reuniting them through electrostatic interactions, van der Waals forces, and/or hydrophobic interactions [81]. This approach is characterized by its ease of operation and minimal membrane loss, making it suitable for mass production. However, it exhibits non-uniform coverage, and the heat energy generated by ultrasound can lead to the loss of cell membrane proteins. Therefore, precise control of ultrasonic parameters is crucial.

Microfluidic electroporation is a new technology developed in recent years to promote the entry of nanoparticles into the cell membrane through electrical pulses. It is composed of five parts, simple operation and outstanding advantages [82]. This technology addresses the shortcomings of the ultrasonic method, which is prone to protein loss, and the extrusion method. And the method realizes high coating rate, retains membrane protein function, high batch production, good parallelism, and has great application prospects [83].

### 3.3 Characterization

**Fig. 2:**
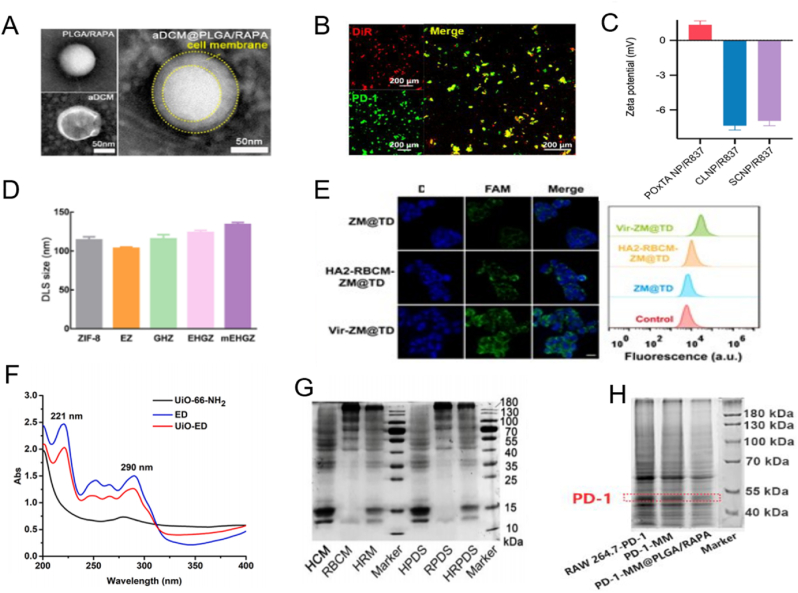
Characterization of BNDS (A) The core-shell structure of BNDS; Copyright 2023 American Chemical Society. (B) Co-localization of cell membrane and nanoparticles; Copyright 2022 American Chemical Society. (C)zeta potential; Copyright 2023 American Chemical Society. (D) Particle size; Copyright 2023 Elsevier. (E) Uptake to cells in vivo; Copyright 2022 Wiley. (F) Fourier infrared spectra of various bonds of BNDS; Copyright 2024 Elsevier. (G) Protein profile of the modified cell membrane; Copyright 2021 Elsevier. (H) Coomassie Brilliant blue with and without modified cell membranes. Copyright 2023 American Chemical Society. (For interpretation of the references to color in this figure legend, the reader is referred to the Web version of this article.)

BNDS can be characterized mainly by size, appearance, quantity, protein content. Since the cell membrane can be distinguished from nanoparticles in terms of surface charge, electron density, protein composition, etc. There are a number of ways that we can determine if the membrane is successfully covered. Transmission electron microscopy is the most direct observation of BNDS. Because the density of cell membrane and nanoparticles is different, the "double layer structure" is particularly obvious under TEM. As is shown in Fig. 2A, the particle size and Zeta potential of nanoparticles are analyzed by Dynamic Light Scattering (DLS) [84]. Since the cell membrane itself has a negative charge, the charge of the nanoparticles will change after successful encapsulation (Fig. 2C) [85]. The particle size of the coated nanoparticles will be slightly larger than that of the uncoated nanoparticles (Fig. 2D) [86]. In fluorescence colocalization (Fig. 2B), the protein on the macrophage cell membrane was marked green by the dye DiR, and the NPs was marked red [87]. The two overlapped, indicating that the cell membrane was successfully coated on the nanoparticle. Cell uptake of BNDS was determined by immunofluorescence and flow cytometry, and the fate of BNDS in vivo was determined by live imaging of small animals and tissue sections (Fig. 2E) [88]. In addition, Fourier infrared spectroscopy showed that after successful membrane encapsulation, the presence of special absorption peaks such as phosphate and amide bonds in the structure of BNDS could indicate the membrane coverage (Fig. 2F) [89]. To verify whether the surface of the cell membrane retains the biological activity of the source cell or the modified cell, SDS-PAGE and western blot are commonly used as protein determination methods in molecular biology. In order to compare the proteoform atlas of source cell membrane cell membrane and BNDS, SDS-PAGE was first performed (Fig. 2G) [90]. Protein composition analysis by SDS-PAGE. The proteins of the original erythrocyte membrane were the same as those of the biomimetic nanosystem [90]. Another study used protein imprinting to identify specific proteins present on macrophage cell-coated nanoparticles (Fig. 2H) [91]. Transfected protein PD-1 was detected on both PD-1-MM@PLGA/RAPA and ordinary cell membranes, and the extent was similar to that of single cell membranes [91]. Similar protein profiles and retention of protein markers are considered reliable indicators of successful nanoparticle entrapment.

In summary, a comprehensive set of procedures for the preparation of BNDS has been evolved, encompassing the isolation of cell membranes, the assembly of cell membrane-coated nanoparticles, and their characterization in recent years. Nevertheless, the approaches of membrane extraction and coating might have an impact on the optimal "biomimetic" functionality of BNDS. Hence, it is essential to guarantee the integrity of the cell membrane and the activity of proteins as possible to ensure the functional exertion of BNDS.

## 4 Application of biomimetic nanoparticles in tumor immunotherapy

### 4.1 Membranes for biomimetic nanoparticle drug delivery system (Table 2)

#### 4.1.1 Red blood cells (RBCs)

Red blood cells are the most abundant and longest-lived cells in human blood. Their main function is to transport oxygen and carbon dioxide for gas exchange in the body [92]. In contrast to nucleated cells, RBCs lack a nucleus and most organelles, rendering the extraction of their membranes relatively straightforward. Consequently, RBC membranes have been extensively utilized in drug delivery systems [93]. The surface of the erythrocyte membrane, which is an important material structure for the maintenance of cell shape and function, can express CD47. CD47 contains five transmembrane regions and a special domain that connects with the inhibitory receptor signal regulatory protein alpha to form the "don't eat me" signal [94]. The interaction enables RBCs to survive in the body for up to 120 days by inhibiting immune elimination by immune cells, thereby prolonging their circulation time, which is also the most prominent feature of the RBCs membrane [95]. Therefore, RBCs camouflage has significant advantages in biofilm-derived nanoparticles.

In recent years, RBCs nanosponges have been rapidly emerging in antimicrobial applications. Neutralizing bacterial toxins is one of the ways to treat bacterial infections and can now be designed for different toxins, but this approach still presents many challenges due to the complexity of bacterial toxins. The RBCs nanosponges can be used as toxin decoys, which can absorb and neutralize a wide range of toxins despite their different molecular structures [96]. *Staphylococcus aureus*, a type of bacteria that causes skin and wound infections, is becoming more resistant as antibiotics become more widely used and stronger strains of methicillin-resistant *Staphylococcus aureus* (MRSA) are on the rise. Biomimetic nanoparticles coated in RBC membranes, which target all bacterial toxins. For example, the erythrocyte membrane was coated on nanoparticle gel through the process of vesicular membrane gelation to prepare the erythrocyte membrane coated nanogel system, which accelerated the drug release curve in the intracellular environment and produced more effective bacteriostasis against methicillin-resistant *Staphylococcus aureus*. In the extracellular environment, the RBC-nanogel can absorb and neutralize the Pore-forming toxins secreted by MRSA bacteria, thereby neutralizing and counteracting the toxicity of the bacteria, and promoting the absorption of the bacteria by phagocytes, showing highly effective inhibition of intracellular MRSA bacteria [97]. Similarly, RBC membrane-coated ciprofloxacin nanoparticles (γ3-RBCNPs) can eliminate *Klebsiella pneumoniae* due to the physiological properties of the RBC membrane that evade immune surveillance, thus prolongs the drug circulation time in the body and minimizes the side effects of antibiotics. In addition, γ3-RBCNPs has a strong targeting effect on the site of infection in vivo, reducing the number of bacteria in infected tissues and inducing apoptosis of infected cells [98].

The surface of red blood cell membranes can undergo modifications to enhance their functionality. RBCs were loaded with hydroxyurea, oxidase, cytosine phosphate guanine oligonucleotide, and the surface of the cell membrane was modified with folic acid to make COF@HGC@FEM. Directs the production of nitric oxide and the consumption of glucose nitric oxide gas therapy is a "green" mode of tumor treatment, folic acid modified erythrocyte membrane can be used to target the delivery of nitric oxide donors to enhance their tumor targeting ability. BNDS can promote the secretion of inflammatory factors such as tumor necrosis factor alpha and interleukin-12 to reduce anti-inflammatory factor levels. Thereby improving the immune microenvironment [99]. Similarly, drug transport through systemic circulation often results in low actual drug concentrations at the tumor site. However, specific ligand-modified erythrocyte membranes can increase the drug targeting ability to the tumor site, addressing this issue. BNDS, prepared by coating the erythrocyte membrane modified with mannitol with dihydrotanshinone, enables the drug to reach the tumor site and release reactive oxygen species (ROS), significantly improving immunogenic cell activity and demonstrating favorable therapeutic effects in liver cancer, breast cancer, colon cancer, and other malignancies [100]. BNDS can be utilized in combination with photothermal therapy (PDT). Following intravenous injection of BNDS, it mediates photothermal therapy (PTT) to deliver a cytotoxic heat dose, inducing cell immunogenic death and improving survival rates [101]. RBCm-IR-Mn was assembled by wrapping near infrared dye IR-780 (IR) in red blood cell membrane, which showed superparamagnetic behavior under quasi-static magnetic field. By controlling the appropriate size and concentration, it can induce apoptosis in advanced tumor cells. In a constructed S180 mouse model, tumor cells were ablated after 5 days of intravenous injection of RBCm-IR-Mn, resulting in an 85 % survival rate for the mice, with no recurrence observed within 120 days [102].

**Fig. 3:**
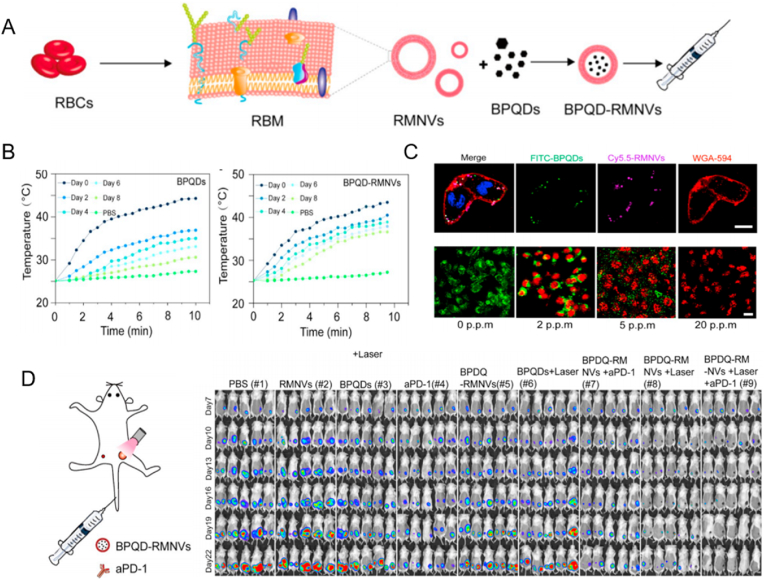
Photothermal therapy and antitumor effects of BPQD-RMNVs (A) Schematic diagram of BPQD-RMNVs synthesis; (B) Comparison of photothermal properties of BPQDs and BPQD-RMNVs at 808 nm laser; (C) The uptake of 4T1 cancer cells and the ability of photothermal elimination of 4T1 cancer cells at 808 nm laser; (D) In vivo bioluminescence imaging of mice with BPQD-RMNV combined with aPD-1 treatment at different time periods under NIR irradiation. Copyright 2019 Elsevier.

In addition, the RBCs membrane coated with black phosphorus quantum dots (BPQD-RMNV) exhibits the capability to diminish immune escape, augment the duration of circulation in vivo, and precisely target tumor site aggregations. Combined therapy with PD-1 can delay the growth of residual and metastatic tumors in vivo, thereby increasing the activity of CD8^+^ T cells and inhibiting the growth of mammary tumors (Fig. 3) [103].

#### 4.1.2 White blood cells

White blood cells are immune cells that play a pivotal role in remodeling the tumor extracellular matrix, facilitating tumor cell migration and invasion, and regulating tumor angiogenesis [104,105]. In response to foreign invaders, white blood cells accumulate in large numbers to eliminate foreign pathogens and dead cells, thereby safeguarding the body against infection [106]. Certain membranes derived from white blood cells, such as neutrophils, dendritic cells, macrophages, and T cells, have been widely used to create biomimetic cell membrane systems. These nanoparticles, coated with white blood cells, inherit their diversity and versatility [107]. They possess cellular self-recognition mechanisms, evade phagocytosis by host cells, and exhibit a shortened circulating half-life in the body. They also inherit receptors on the surface of white blood cells, giving them the advantage of preferentially binding to diseased sites [108].

By blocking immune checkpoint PD-1/PD-L1 to become an effective strategy for the treatment of tumors. A white cell membrane coated with glycyrrhetinic acid has been developed, and the resultant GCMNPs, in conjunction with ferrite, promote ferroptosis through the Fenton reaction. The combination of iron oxides can enhance the blocking of PD-1/PD-L1, thus activating T cells. For improving the immune response of T cells in leukemia and colorectal tumors [109]. Similarly, the T cell membrane is coated with polylactic-glycolic acid copolymer (PLGA) to prepare the nanoparticle TCMNP, which uses the T cell membrane to target tumors and kill cancer cells by releasing anticancer factors and clearing TGF-β1 and PD-L1, thereby reactivating cytotoxic immune cells. TCMNPs has shown higher therapeutic efficacy in the treatment of melanoma and can also block PD-1/PD-L1 signaling, which has a significant effect on inhibiting tumor growth [110].

In addition, TCMNP is used after modification on the surface of cell membranes. One of the reasons why MHC-1 deficiency causes the dysfunction of cancer cell antigen presentation is associated with poor prognosis and metastasis in many cancers. In mouse models of colon and pancreatic cancer, injecting LCL616 into mice observed a 42 % reduction in tumor volume and an increase in T cell density. LCL161 modified macrophage cell membranes to form LCL161-loaded macrophage membrane decorated nanoparticle (LMN), which significantly increased the levels of inflammatory factors in tumors. In addition, LMN promotes the endocytosis of CD47 on the surface to block the "don't eat me" signal generated by the tumor and induce effective communication between immune cells [111]. Similarly, DSF/Cu^2+^ complex was prepared to induce copper death in the treatment of triple-negative breast cancer (TNBC), so that high levels of Cu^2+^ in tumor cells can induce endoplasmic reticulum stress and promote the occurrence of immunogenic cell death (ICD) in tumor cells. DSF/Cu^2+^ was further coated with PD-1-expressing T cell membrane to increase its targeting function. Its expression of PD-L1 can significantly enhance cellular immune escape, thereby allowing CuX-P to accumulate in tumor cells, induce copper apoptosis and immune response, and inhibit the occurrence of TNBC [105].

**Fig. 4:**
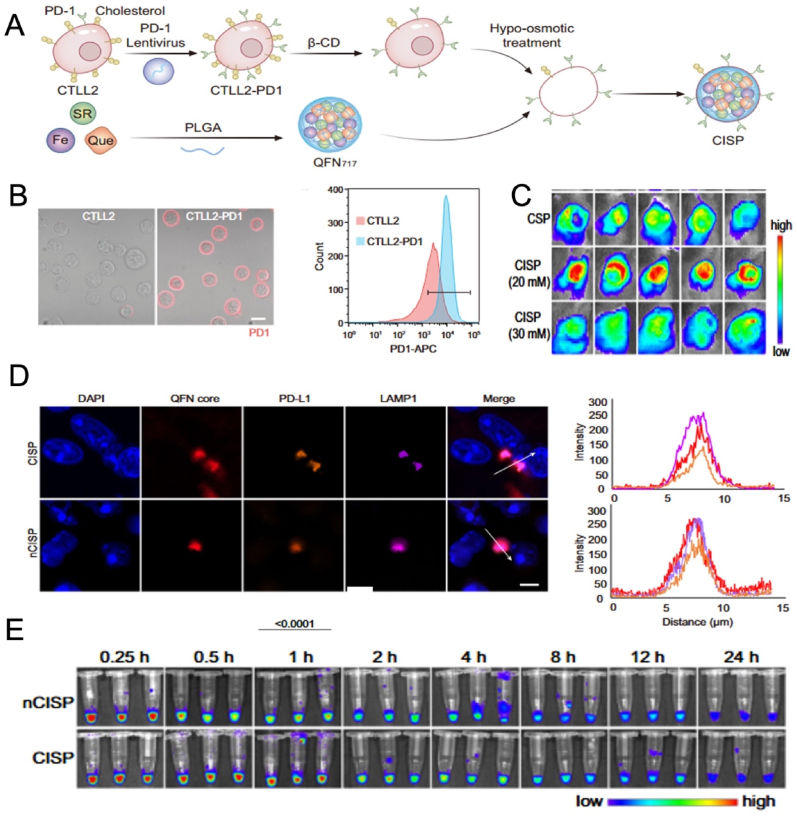
CISP targets tumors in vivo and inhibits clearance of CISP by monocytes in the blood. (A) Schematic diagram of the preparation of CISP; (B) PD-1 expression on CTLL2 cells; (C) Fluorescence images of biomimetic nano with PD-1 expression (CISP) and biomimetic nano without PD-1 expression (CSP) on B16F10 tumors; (D) Uptake of cell membranes with or without cholesterol (nCISP, CISP) into B16F10 tumors; (E) Cell membranes with or without cholesterol (nCISP, CISP) inhibit clearance of CISP by monocytes from the blood. Copyright 2023 Nature.

Furthermore, it can be used in combination with photothermal therapy based on cell membrane modification. The expression of PD-1 on T cell membrane increases its tumor targeting ability. β-CD treatment of live CTLL2-PD1 cells (CISP) reduces the cholesterol content in the cell membrane. The uptake of CISP by monocytes in the blood is reduced by about 50 %, indirectly enhancing its tumor targeting ability. The combination of photodynamic agents and STING agonists can increase tumor targeting ability by 2 times (Fig. 4) [112]. The dendritic cell (DC) membrane-encapsulated photosensitizer (AIE) facilitates antigen presentation. Due to the presence of the DC membrane, the accumulation of AIE at the tumor site was 1.6-fold higher than that in the control group. Once the photosensitizer reached a sufficient concentration, photodynamic therapy (PDT) was administered. The combination of AIE and PDT significantly enhanced T-cell proliferation and activation, leading to a marked reduction in the size of in situ tumors [113].

#### 4.1.3 Cancer cell

In the metastatic process, cancer cells will face problems such as insufficient nutrition and hypoxia. The survival of tumor cells hinges on their ability to adapt metabolic activities to fulfill energy demands during progression [114,115]. Cancer cells express both "self-labeling" and "self-recognition" molecules, which synergize to enable immune escape and homologous binding capabilities, thereby overcoming the hurdles of immune clearance and non-specific binding [116,117]. BNDS disguised by cancer cell membranes are recognized as endogenous cancer cells. Due to the adhesion and proteins on the cancer cell surface, they exhibit strong targeting ability towards the same cell line, enabling the precise delivery of cancer cell-coated nanoparticles to the lesion site [118]. Homologous targeting of tumor cells is dependent on interactions with T antigen and beta-galactosid-binding proteins and galectin-3, as well as adhesion proteins on the surface of tumor cell membranes. Therefore, tumor cell membranes possess a significant advantage as components of BNDS [[119], [120], [121], [122]].

Paclitaxel-loaded liposomes (LP@BG@CCM) coated with mouse breast cancer membrane 4T1 were prepared by layer upon layer encapsulation. After intravenous injection, compared with the control group, the apoptosis rate of lung cancer cells was increased. The appearance of the lungs of mice was significantly improved, the lung tissues of mice were obviously restored to normal and the weight was reduced. Additionally, immune factors such as CD4+/CD8a^+^ T cells and cytokines like TNF-α, IFN-γ, and IL-4 in the lungs were notably enhanced. The release of surface LP can successfully target the effects of lung, cancer cell killing, and immune stimulation [123]. A biomimetic nanoparticle system synthesized from artificial antigen-presenting cells (aAPC) CD-MnOx@CM has been developed to enhance pulmonary metastasis immunotherapy. By catalyzing the decomposition of H_2_O_2_ into oxygen in tumor to regulate tumor microenvironment, aAPC significantly inhibits the development of lung metastasis, prolongs the survival of B16-F10 melanoma metastasis model, and provides a new approach for tumor metastasis therapy [124].

The nanoparticles were prepared into nanoparticle vaccines. In a study, CaP-NPs was prepared by coating B16-OVA tumor cell membranes with nano-sized calcium pyrophosphate. Biofilm proteins served as tumor-specific antigens, and calcium pyrophosphate nanogranules exhibited excellent biocompatibility, improving the efficiency of antigen delivery and uptake. This activated the NLRP-3 inflammasome and elicited various T cell responses, achieving effective delivery to APCs and activating immune cells [125].Similarly, there is a nano-vaccine PTh/MnO2@M that utilizes photoelectric conversion in near-infrared light to produce reactive ROS, inducing local micro-inflammation, promoting antigen transfer to T cells, eliciting anti-tumor immunity, and effectively inhibiting melanoma growth in mice with low biological toxicity and good safety [126].

**Fig. 5:**
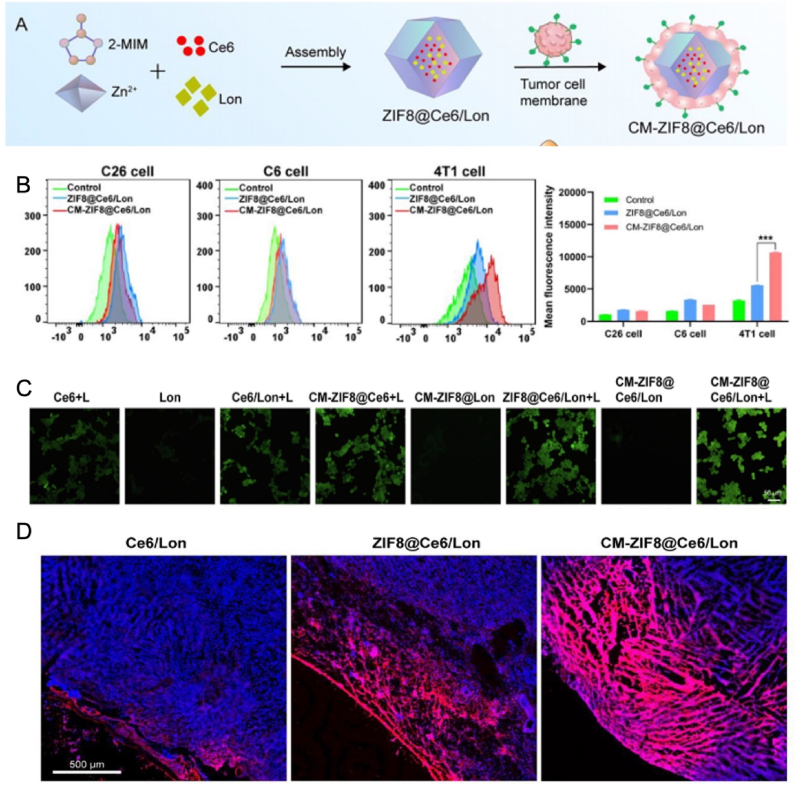
CM-ZIF8@Ce6/Lon photodynamic properties, in vivo targeting, and biodistribution. (A) Preparation of CM-ZIF8@Ce6/Lon; (B) Flow cytometry analysis of 4T1 cancer cells against CM-ZIF8@Ce6/Lon through different cancer cell lines; (C) In vitro ROS production induced by various treatments with or without laser irradiation; (D) Determination of distribution and permeability in tumor tissue by CLSM imaging of frozen tumor sections. Copyright 2023 Elsevier.

Immunotherapy and photothermal therapy (PTT) are used in combination. A biomimetic nanosystem was developed to inhibit TNBC tumor growth. The system consists of polylactic-glycolic acid copolymer (PLGA) of IR-II Ag2S QDs, PTX and PD-L1 inhibitors. In mouse lung metastatic tumor models, AgPP@P@M and PTX showed excellent photothermal conversion ability and activated ICD under the action of infrared [127]. Similarly, tumor cell membranes were disguised with ZIF-8, Ce6, and Lon to prepare CM-ZIF8@Ce6/Lon cooperative PDT amplified immunotherapy, which exhibited a marked specific homotypic targeting to 4T1 cells. Laser irradiation induced the production of ROS, leading to tumor cell apoptosis. Due to the modification of cell membranes, this homotypic targeting can penetrate deeper into the tumor tissue (Fig. 5) [128]. Glioblastoma-derived nanoparticles (MDNP) were prepared by coating photosensitizers and therapeutic drugs onto the surface of glioblastoma cells. These MDNPs exhibit homologous targeting accumulation and prolonged circulation in vivo, enabling them to effectively cross the blood-brain barrier. Upon reaching the tumor site, they generate reactive oxygen species (ROS) to induce apoptosis of tumor cells. The combination with phototherapy facilitates precise, wireless, and continuous anti-tumor treatment [129]. DOX, IR-780, and ZIF-8 were encapsulated into nanoparticles, which were subsequently coated with tumor cell membranes to form D/INPs@CM. Leveraging the homologous targeting properties of tumor cell membranes, D/INPs@CM can specifically target tumor cells within the bone marrow microenvironment. Upon reaching the target site, the nanoparticles release DOX and IR-780, enabling a synergistic effect between chemotherapy and photothermal therapy. This approach significantly inhibits tumor proliferation and effectively suppresses multiple myeloma [122].

Biomimetic nanoparticle Cu_2_-xSe NPs was coated with CD6-expressing tumor cell membrane and formed CS-J@CM/6 NPs with the antidepressant Paxil (PX) after modification of tumor cell membrane surface. PX increased the expression of S1PR1 on T cells, thereby augmenting the number of T cells in the tumor. A small molecular inhibitor JQ1 not only reduces the expression of PD-1 and TIM-3 on T cells, but also downregulates the expression of TET2, improving the function and activity of T cells to activate the immune response through the synergistic action of multiple receptors [130]. Situ dual glycan was bound to tumor cell membrane by electroporation. Three immunotherapy drugs (TEID) are wrapped in a shell of hyaluronic acid to identify the tumor site by CD44 on the surface of the tumor cell membrane. Growth factors released by TEID can be reshaped on the tumor cell membrane to secrete cytokines for immune killing, thereby reducing immunosuppression [131].

#### 4.1.4 Platelets

Platelets are cells that play a role in the process of primary hemostasis. They are derived from mature megakaryocytes within the bone marrow. Their main role is hemostasis and the formation of blood clots [132]. Platelets are participants in many pathophysiological processes, including inflammation, the formation of atherosclerosis, tumor growth and metastasis, and antimicrobial host defense. Due to the expression of CD47 on their surface, platelets possess a circulatory half-life of approximately 30 h, enabling them to extend their systemic circulation time. Platelets act as targeted anti-tumor agents under the action of a variety of surface receptors and glycoproteins, such as podoplanin, GPPIb-IX-V complex, GPVI, and integrin αIIbβ3. The platelet-derived membrane still retains its original adhesion function [[133], [134], [135]].

Intracorumoral administration of platelet-coated nanoparticles (PNP-R848) significantly enhanced local immune activation. BNDS promoted the activation of APC in lymph nodes in a mouse model of colorectal cancer, and was equally effective in a 4T1 mouse model of triple-negative breast cancer [136]. The bionic nanosystem DR@PLip, coated with adriamycin (DOX) and ginsenoside (Rg3), utilizes the natural adhesion effect of the platelet membrane on AML cells. The combination of these two drugs significantly enhances the ICD effect, thereby stimulating the immune response and exhibiting a favorable therapeutic effect in the treatment of acute myeloid leukemia [137]. Glutathione (GSH) plays a key role in the treatment of cancer when immunotherapy and radiotherapy are used together. A core particle consisting of MON and 2-DG was designed to be coated with platelets membrane. The consumption of glutathione can be induced to enhance the effect of radiation therapy. The MON and GSH reactions release 2-DG and consume GSH content in the first step. The subsequent release of 2-DG inhibits glycolysis, eventually depleting GSH further. The double consumption of GSH and the synergistic treatment of radiotherapy caused the immunogenic cells to die, and the DC cells and CD8^+^T cells were also activated [138].

**Fig. 6:**
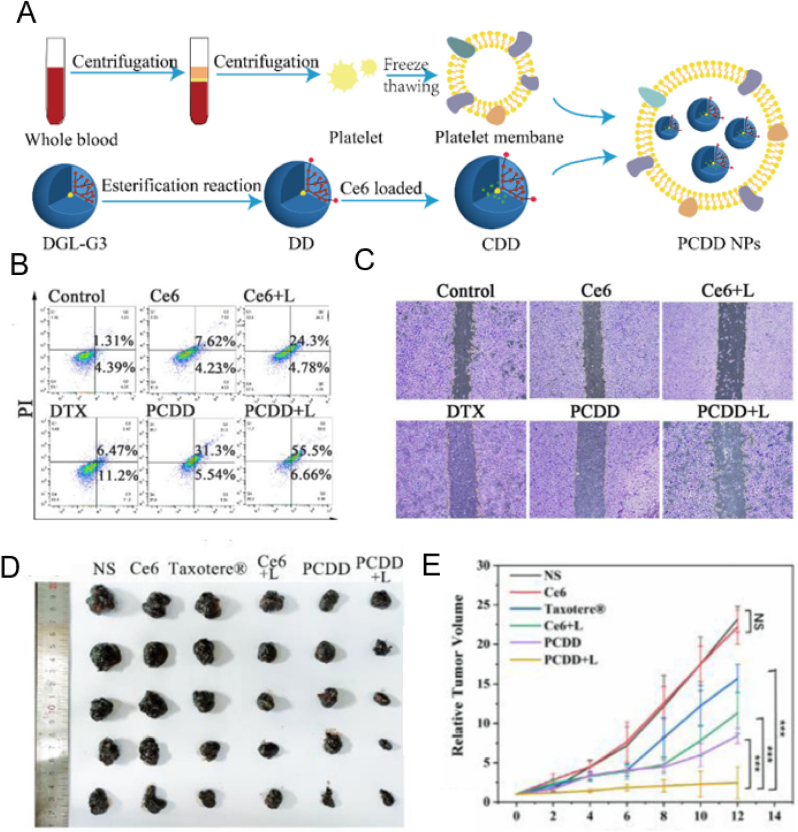
In vivo targeting and anti-distant tumor and metastasis assays. (A) Preparation process of PCDD NPs; (B) Map of apoptosis; (C) Pictures of colony scratch; (D) Tumor size; (E) Tumor volume. Copyright 2022 The Royal Society of Chemistry.

Novel platelet coated nanoparticles (PCDD NPs) combined with chemo-photodynamic therapy and immunotherapy were constructed. PCDD had the strongest killing ability on tumor cells. After near-infrared treatment, the number of colonies in PCDD group was significantly reduced. There was strong targeting in B16F10 tumor-bearing mice. The tumor inhibition rate was 92.0 %(Fig. 6) [139]. Platelet membranes were coated with paclitaxel to prepare PM-NP/PTX, and anti-PD-1 antibodies were released at the tumor site. Antitumor efficacy was demonstrated in a retumor breast cancer model [140].

#### 4.1.5 Exosomes

Exosomes are extracellular vesicles released by all cells and are composed of protein, nucleic acid, bioactive molecules, and lipid extracellular vesicles. They range in size from 40 to 100 nm. The contents of exosomes are inherited from the source cell, so specific sites can be targeted by selecting the source of exosomes [141]. This explains why they are playing an important role in immune escape, cell linkage, and specific cell uptake. Exosomes have a good role in biomimetic cell membranes due to their endogenous, immunocompatible, nanoscale size [142].

In order to treat allergic asthma in mice, the exosome membrane of M2 macrophage cell membrane coated with EM-PLGA@Dnmt3aos obtained by nanoparticles and inculcated BNDS can significantly improve asthma in mice, significantly reduce lung inflammation, and significantly reduce the retention time in vivo for more than 48 h and the proportion of inflammatory cytokines [143]. The existence of blood-brain barrier is hindered in the treatment of glioblastoma. Mouse glioblastoma cell line BV2 is now used as an exosome to be coated with doxorubicin to make Pep2-Exos-DOX. DOX is relatively assembled in the brain, has good penetration behavior, and shows the best anti-glioma activity [144]. Through co-incubation, the exosomes of macrophages were coated with iron oxide nanoparticles to obtain ESIONPs@EXO, which effectively inhibited pathological angiogenesis, weakened the production of pathological retinal blood vessels, and the formation of pathological blood vessels in melanin, providing an effective idea in the treatment of pathological angiogenesis [145].

**Fig. 7:**
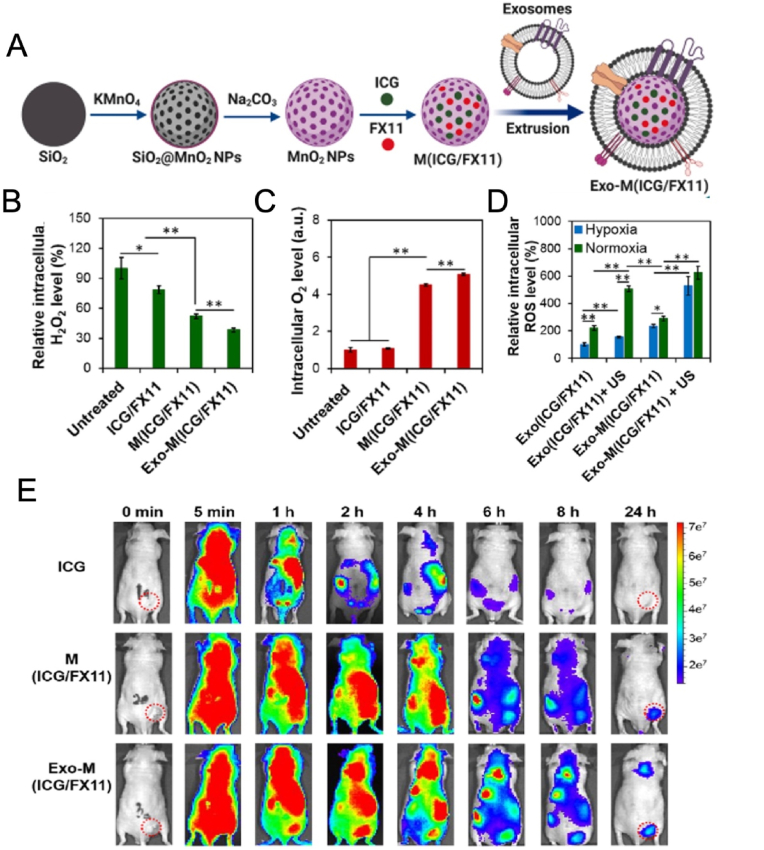
In vivo tumor accumulation and anti-tumor effect of Exo-M (ICG/F X11) under hypoxic conditions. (A) Preparation of Exo-M (ICG/F X11); (B) Quantification of intracellular H_2_O_2_ levels in MCF-7 cells after various treatments; (C) Quantification of intracellular O_2_ levels in MCF-7 cells after various treatments; (D) Quantify intracellular ROS levels in MCF-7 cells incubated with various samples under normoxic and hypoxic conditions; (E) In vivo biodistribution of MCF-7 tumor xenograft mice at different time intervals after vein injection. Copyright 2023 Elsevier.

The use of ultrasound ratio (US) and sonodynamic therapy (SDT) has a good effect in the treatment of tumors. It overcomes the conventional poor biocompatibility and hypoxia of SDT in the tumor microenvironment, catalyzes H_2_O_2_ to produce O_2_ to alleviate tumor hypoxia, and live sound significantly increases the intracellular ROS level after US irradiation. It has obvious targeting effect on tumors and good biocompatibility (Fig. 7) [146]. Due to the short half-life of ultrasound contrast agents in the blood system and the difficult modification of the surface of microvesicles, exosome membranes were used as transport carriers of ultrasonic photosensitivists dihydroporphyrin, and the nanoparticles Ce6-Exo-MBs was prepared and delivered to tumors. Since PDT delayed tumor growth and was combined with anti-PD-L1 antibodies, compared with single therapy, Ce6-EXO-MBS was prepared and delivered to tumors. The tumor weight and mean survival cycle of the mice were significantly improved [147].

#### 4.1.6 Hybrid cellular

Compared to a single cell, hybrid cellular membranes possess the capability to inherit biological functions from their source cells, such as cell targeting and extended circulation, thereby demonstrating remarkable efficacy and safety profiles. Owing to technological advancements, it is now possible to fuse two or more cell membranes [[148], [149], [150]]. This offers the potential to change the nature of nanoparticle and facilitate targeted drug delivery. Hybrid cellular not only allows for the simultaneous combination of properties from two different parental cell membranes but also enables the integration of multiple functions [151].

**Fig. 8:**
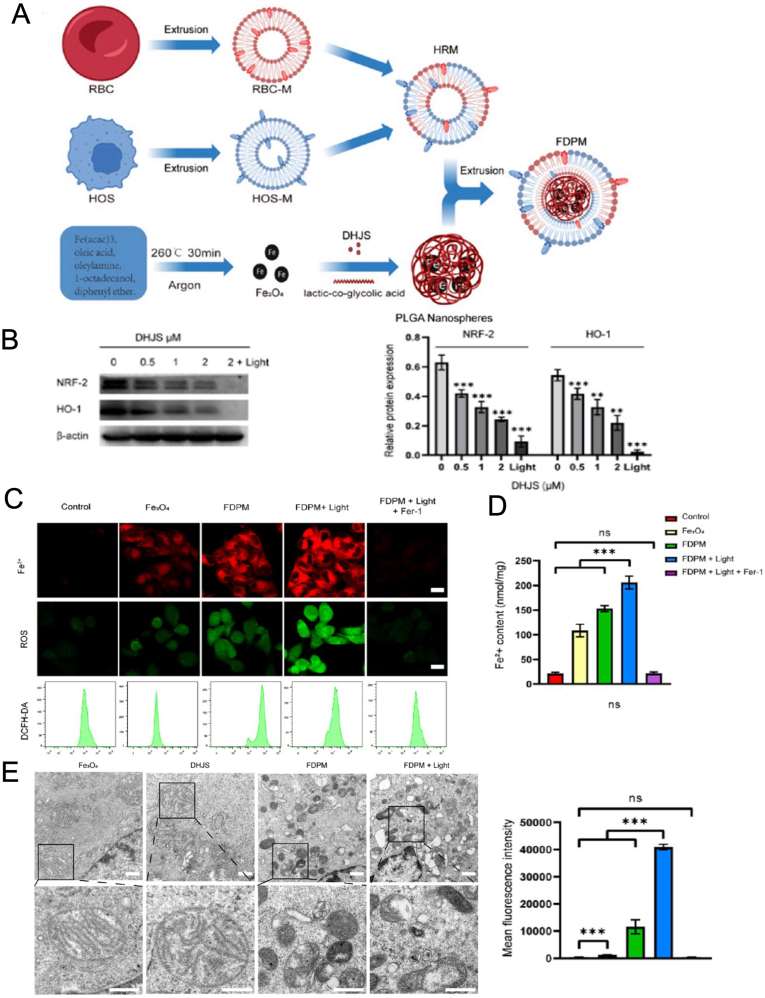
Triggers lethal ferroptosis in HOS cells. (A) Preparation of Fe_3_O_4_-DHJS@HRM nanoparticles; (B) Effect of DHJS nanoparticles on the overall expression of NrF2-related pathways in HOS cells; (C, D) Fe^2+^ and ROS in HOS cells after different treatments were detected by CLSM or FCM using fluorescent probes; (E) Morphological assessment of mitochondria after treatment of HOS cells with various nanoparticles. Copyright 2023 Multidisciplinary Digital Publishing Institute.

Nanoparticles were assembled from the ultra-small light therapeutic agent Black phosphorus quantum dots, the chemotherapy drug paclitaxel (PTX) and the immunomodulator polymetformin (PM). BPP@RTL NPs was prepared by fusing the cell membranes of cancer cells and red blood cells, which coated with nanoparticles. The physiological characteristics of the two source cells were preserved. When combined with an anti-PD-L1 antibody and subjected to NIR laser irradiation, the survival time of 4T1 tumor-bearing mice was significantly prolonged, and the metastasis of breast tumors was inhibited [152]. A bionic hybrid cell membrane camouflaged by PLGA-loaded Fe_3_O_4_ and DHJS was prepared to treat osteosarcoma. Cancer cells can prevent ferroptosis by overexpressing NRF-2-related antioxidant systems. When Fe_3_O_4_ nanoparticles synergized with DHJS (a probe for ROS generation), they could antagonized the expression of Nrf-2, thereby inducing ferroptosis in tumor cells. ROS and Fe^3+^, as significant markers of ferroptosis, were significantly increased. The potential changes and mitochondrial damage further indicated that the synthesized nanoparticles exhibited promising therapeutic effects on Osteosarcoma (Fig. 8) [153]. To treat colorectal cancer and overcome the issue of low biocompatibility, red blood cells were hybridized with 293T cells expressing PD-1 to prepare nanoparticles CDDP@HZGG@RPDM. For tumor tracking in vivo, the growth of colorectal cancer tumors in CT26 tumor-bearing mice was significantly inhibited on the basis of combination therapy [154]. There is also a tumour-specific nanovaccine FCM@4RM, which is composed of Fe (II), deoxynucleotide (CpG), and metformin (MET) coated with fusion cells of tumor 4T1 cells and RAW264.7 macrophages. Metformin is an inhibitor of PD-L1, which can block PD-L1 signal, restore the immune response of T cells, and significantly inhibit tumor growth and recurrence [155].

In summary, different types of cell membranes possess distinct characteristics. Red blood cell membranes can significantly prolong circulation time in the bloodstream and evade immune clearance. Cancer cell membranes enhance homologous targeting, while white blood cell membranes not only improve tumor targeting but also augment immune regulatory functions. The "biomimetic" structure retains the surface properties and functional attributes of the source cell membrane. For various types of tumors, BNDS derived from different cell membranes can be synergistically combined with diverse therapeutic modalities to enhance drug delivery [156,157]. The multi-functionality, multi-targeting capability, biocompatibility, and extended circulation time of BNDS underscore its vast potential in tumor immunotherapy.

### 4.2 Engineered modifications of cell membranes

**Fig. 9:**
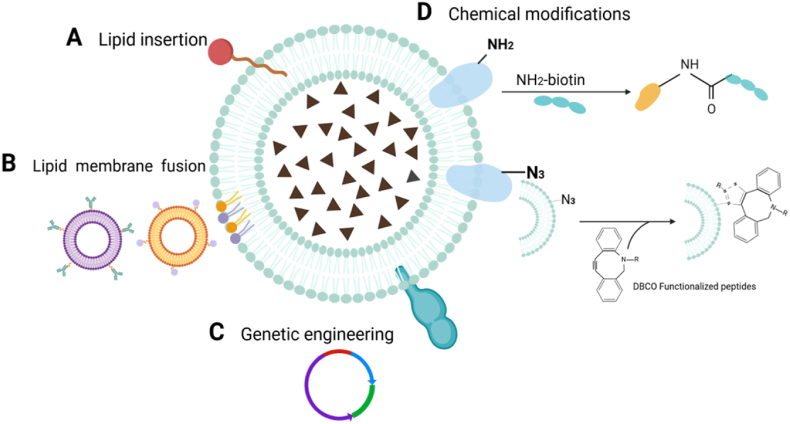
Engineering modification of cell membrane. Created with BioRender.com.

The function of cell membrane is mainly determined by the functional proteins on the surface. In order to break through the limitations of natural cell membrane in disease treatment and be used for more accurate targeted research and treatment, engineering modifications of the cell membrane are necessary to enhance its biological activity. Generally, membrane modifications encompass physical modification, chemical modification, genetic modification, and cell membrane fusion, among others (Fig. 9).

#### 4.2.1 Physical modification

Physical modification is a relatively benign process that leverages the fluidity of the lipid bilayer while preserving the activity of the proteins on the surface of the protocell membrane. Lipid insertion is a simple and stable modification that is commonly used to spontaneously embed into the phospholipid bilayer structure through hydrophobic action to provide a stronger binding force.

**Fig. 10:**
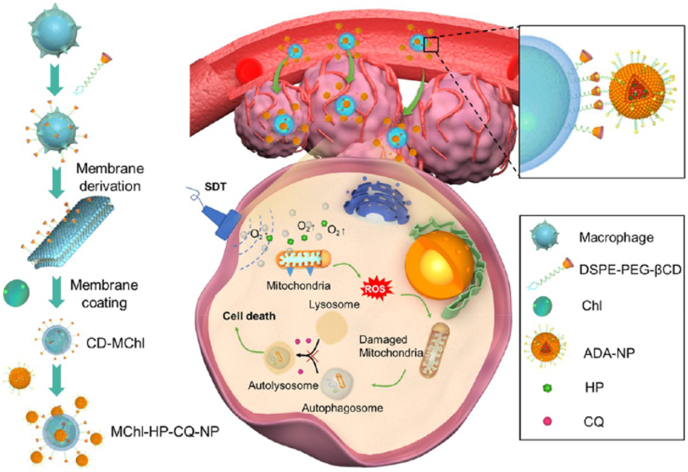
Physically, in the form of lipid insertion, β-CD modifies the surface of macrophages to induce ICD. Copyright 2023 American Chemical Society.

Gao et al., due to the rigid membrane structure of MChl (macrophage), hinders the surface modification of β-CD. Therefore, MChl was modified with β-CD through lipid insertion to create MChl-NP [158]. The modified macrophage cell membrane can enhance the sonodynamic therapy (SDT), promoted the liposomes carrying bidrug to target the melanoma site together, and improved the biocompatibility. Furthermore, macrophages sustainably produced oxygen to alleviate features associated with tumor hypoxic sites, inhibited HIF-1α expression in B16 cells, and enhanced the SDT efficacy of MChl-HP-NP, enabling T cell activation and reversing the tumor immune microenvironment (Fig. 10) [158]. Liu et al. modified RBC membrane with hyaluronic acid by means of lipid insertion, loaded with CS-6 nanocomplex [159]. Erythrocyte membrane can significantly improve the aggregation of drugs at tumor sites, and the modified HA binds to CD44 receptors on breast cancer cells, further enhancing tumor site targeting and increasing immune escape ability by more than 60 %. The blood circulation time was extended to 10 h, with no toxic or side effects observed on normal tissues.

#### 4.2.2 Chemical modification

The nanoparticle particles are covalently bound to the cell membrane by primary amine (-NH2) and mercaptan residues (-SH) of the surface protein and hydroxyl residues (-OH) of the polysaccharide. The chemical modification on the cell membrane enables the cell to endow more chemical groups, thus giving the nanoparticles a wider range of functions. However, the active protein on the cell membrane is easily destroyed during the modification process, so the reaction conditions need to be controlled.

**Fig. 11:**
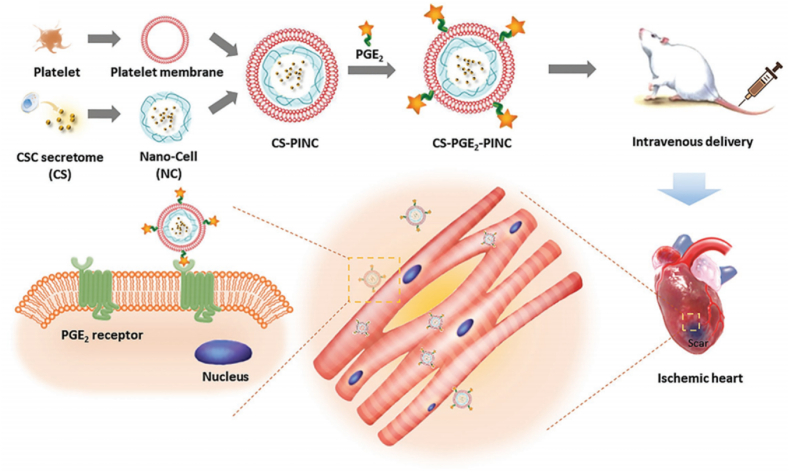
Chemically, GelMA and T-NNPS nanoparticles are linked with an amide bond to form GM@T-NNPs. Copyright 2018 Multidisciplinary Digital Publishing Institute.

Zhou et al. connected polylactic-glycolic acid copolymer nanoparticles (T-NNPs) coated with growth faction-β1 (TGF-β1) loaded neutral particle cell membrane to the surface of methacrylate gelatin anhydride microspheres (GM) via an amide bond [160]. The amine group could form an amide bond with the carboxylic acid group. The formation of the amide bond formed a stable nanoparticle - microsphere complex (GM@T-NNPs). Similarly, Li et al. took magnetized Fe_3_O_4_ as the nanocore and used cancer cell membranes expressing CD205 [161]. The cancer cell membranes were designed to be azide-modified, providing a mild and efficient chemical site, and subsequently modified with dibenzocycloctyne modified (DBCO modified) anti-CD205. The CD205-expressing cell membranes were preferentially recognized by CD8^+^ dendritic cells (DCS), which are mainly concentrated in the lymph nodes. This recognition stimulates the proliferation of CD8^+^ T cells and T cells, thereby achieving potent anti-cancer efficacy and safety. Su et al. PGE22 reacts with the amine group of the platelet membrane to obtain the functionalized platelet membrane of PGE, which enhances the cardiac function and promotes angiogenesis (Fig. 11) [162].

#### 4.2.3 Genetic modification

**Fig. 12:**
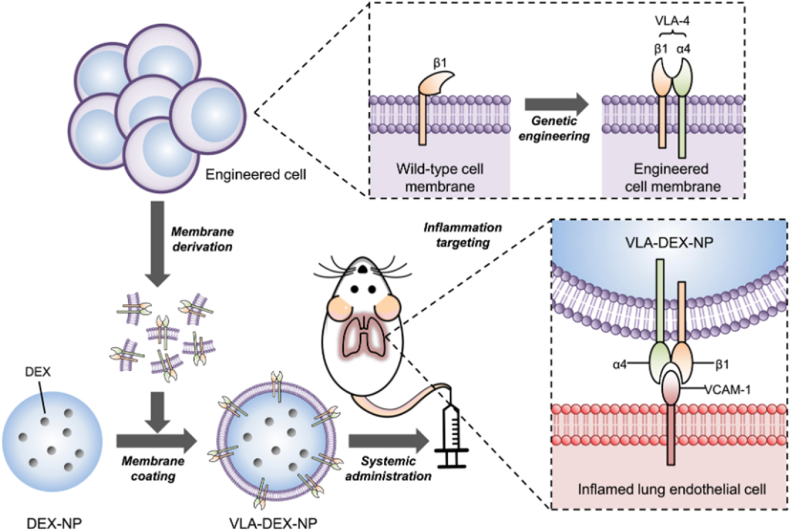
Genetic modification. Expression on the cell membrane surface VLA-4 protein was used to target pneumonia. Copyright 2021 American Association for the Advancement of Science.

The proteins expressed on the cell membrane are determined using nucleic acids. And genetic modification is to introduce foreign DNA and RNA into cells through viral transfection, non-viral transfection, electroporation transfection, etc. Through transcription translation, the required proteins or peptides are expressed on the surface of the cell, which is then convenient to camouflage the kernel. It is now used in tumor vaccines and immunotherapy. However, the genetic modification steps are intricate, and ensuring stable expression of the required proteins remains challenging, thereby limiting its widespread application. Zhang et al. transfected the cell membrane of mouse breast cancer cells 4T1 by lentivirus packaging, and made 4T1 cells express PD-1. Cancer cells expressing PD-1 showed synergistic enhancement of therapeutic effect in subcutaneous and lung metastasis models of breast cancer, blocking the function of immune checkpoint PD-1/PD-L1 ligand and activated T cells [163]. In vitro expansion of CDT-induced ICD cascade, the combination increased the immunogenicity of TME, and the tumor had good targeting. Similarly, Park et al. prepared the anti-inflammatory drug dexamethasone into nanoparticle preparations for the treatment of pneumonia. At the cellular level, inflamed endothelial cells lead to upregulations such as vascular cell adhesion molecule-1 (VCAM-1) or intercellular adhesion molecule-1 (ICAM-1). To enhance targeting functionality and modify the tumor immune microenvironment, the surface of white blood cells was stably expressed with VLA-4 protein through the packaging of genetically engineered lentivirus, enabling better targeting of inflamed lungs (Fig. 12) [164].

In addition, cell membranes can express a variety of proteins, such as CD47 [165], a transmembrane protein expressed in a variety of tumor cells, binds to SIRPα to send a "don't eat me" signal to avoid immune clearance. Blocking this pathway promotes uptake of the drug by tumor cells. Calreticulin can bind to the receptor on the surface of DC to send a "eat me" signal to promote antigen phagocytosis and activate natural immunity. CD47 and CRT are a group of positive and negative signaling molecules that improve cell immunogenicity. The nanoparticle vaccine was prepared by transfecting B16F10 tumor cells with two target genes by non-viral transfection. The nano-vaccine was combined with PD-L1 antibody to further block the inhibition of tumor tissue on T cells [166].

Overall, the modification of cell membrane surfaces via physical, chemical, and biological methods can confer new functionalities to the cell membrane. The modified membranes, building upon their original properties, exhibit enhanced tumor targeting, improved biocompatibility, or extended circulation time in the bloodstream. Although the modified membrane system may require further clinical validation, the targeted, therapeutic, and imaging technologies, pH, temperature, enzyme, and other responsive modifications are all likely to become future development trends.

## 5 Prospects and challenges

Immunotherapy has achieved remarkable success in cancer therapeutics. However, its clinical translation remains constrained by persistent challenges including drug targeting precision and insufficient immunogenicity. To address these limitations, there is an urgent need for rational biomaterial design to enhance both the safety and targeting efficacy of therapeutic agents. In recent years, the rapid advancement of BNDS has provided novel strategies for tumor immunotherapy. Therefore, this article provides an overview of the immunological characteristics of the tumor microenvironment and current therapeutic classifications. We subsequently introduced commonly used methods for preparing BNDS. Finally, the latest research on the modification of membranes and membrane surfaces of various types of biomimetic nanoparticles were reviewed. The application of BNDS in tumor immunotherapy is currently in a phase of rapid development, with multiple clinical trials demonstrating their potential in cancer treatment (Table 3). Those clinical trials currently span various tumor subtypes and disease stages, indicating significant research interest and promising clinical application prospects.

**Table 1:** The disadvantage and advantage of cell membrane coating method.

Cell membrane coating method	Specific methods	Disadvantages	Advantage
Co-extrusion [80]	A mixture of cell membranes and nanoparticles is prepared by extrusion back and forth through polycarbonate using an extruder	The particle size is uniform and the activity of cell membrane protein can be maintained	The operation is time-consuming and laborious, the sample loss is large, and it is not suitable for mass production
Ultrasonic method [98]	A mixture of cell membrane and nanoparticles is prepared by combining them with non-covalent bonds at a certain frequency	Small sample loss	Non-uniform particle size and easy inactivation of cell membrane protein
Microfluidic electroporation [82,83]	The mixture of cell membrane and nanoparticles is passed through the electroporation area by means of electrical pulse, and finally collected in the chip for preparation	It is safe and reliable and can be prepared in large quantities	Cell membrane proteins are easily inactivated

**Table 2:** Membranes for biomimetic nanoparticle drug delivery system.

Types	Core pellets	Wrapped medication	Site of action	Mechanism of action
Red Blood Cells	Nanogel	Antibiotics	bacteria	Effectively neutralize MRSA-associated toxins in the extracellular environment [112]
	PLGA	Ciprofloxacin	lung	Gamma-3 peptides target the infected lesion site ICAM-1 to kill Klebsiella [114]
	COF	GOx, Hu, CPG	Triple negative breast cancer	GOx directed catalytic reaction to consume glucose for starvation therapy [99]
	PLGA	Plga (PLB), dihydrotanshinone I (DIH)	Hepatocellular carcinoma (HCC)	DIH produces ROS, which mediates ICD activity and reverses immunosuppressive TME [100]
	Fe3O4	IR-780	sarcoma	ablative regime,PTT induces immune response [102]
	BPQD	Black phosphorus (BP)	Breast CANCER	increased the infiltration and activity of CD8^+^ T cells in the tumor [121]
White blood cells	PLGA	glycyrrhetinic acid	Leukemia, colorectal cancer	promote a Fenton reaction and induce ferroptosis [109]
	PLGA	anticancer drugs	Melanoma	release anticancer molecules and inducing Fas-ligand-mediated apoptosis [110]
	LBN	LCL161	Triple negative Breast Cancer (TNBC)	Block CD47-mediated phagocytic inhibition and inhibit the growth of MHC-I deficient TNBC tumors [111]
	CuX-P	DSF, Cu	Triple Negative Breast Cancer (TNBC)	Induces cell apoptosis, resulting in increased CRT expression and promotes recognition and activation of tumor-associated antigens [166]
	PLGA	SR-717	Colon Cancer	Increase the permeability of cell membrane and decrease the non-specific binding between cells [112]
Cancer Cells	BGs	Paclitaxel (PTX)	Lung cancer	Delivery of PTX, CD4+/CD8a + T cells, etc. is improved [123]
	MnOx	Au	Melanoma	Enhanced expansion and activation of CD8^+^ cytotoxic T cells and dendritic cells within the tumor, thereby regulating TME [130]
	CaP	calcium pyrophosphate	Lymph nodes	achieve effective payload of antigens and to be efficiently delivered to APCs for activating specific cellular immunity [125]
	MnO2	Polythiophene (PTH)	melanoma	promote APC maturation and autologous tumor antigens presentation [126]
	PLGA	PTX	Triple negative Breast Cancer (TNBC)	PTT induced ICD [127]
	ZIF8	Ce6	Triple negativBreast Cancer (TNBC)	ICD induction by PDT [136]
	Cu2-xSe	PX	Glioblastoma (GBM)	Decreased expression of PD-1 and TIM-3 on T cells and PD-L1 on tumor cells [130]
	HA	SLO-Gal, SLO-NEU	Breast cancer	Activation promoted NK cells and weakened immune cell inhibition [131]
Platelets	PNP	R848	Colorectal cancer, breast cancer	Promotes strong activation of APC in drained DLN and increases immune infiltration [138]
	DR	DOX, Rg3	Acute myeloid leukemia	Activated T cells are combined with PD-L1 antibodies to enhance ICD [137]
	MON	2-DG	Breast cancer	Double GSH consumption that promotes immunogenic cell death [138]
	DD	Ce6	Melanoma	PDT induced ICD [139]
	NP	PTX	Breast cancer	Enhancement of tumor selective ICI accumulation [140]
Exosome	PLGA	Dnmt3aos	pneumonia	Promotes gene silencing and reduces the degree of airway inflammatory cell infiltration [143]
	Pep2-Exos	DOX	Brain	Through endocytosis, through the blood-brain barrier [144]
	ESIONPs	EXO	Pathological vessel	Inducing iron death [145]
	MnO2 NPs	ICG	Hypoxic tumors	Expending energy and relieving hypoxia enhances SDT in hypoxic tumors [146]
	Exo-NPs	Ce6	Enhanced ultrasound contrast agent	PDT induces ICD [147]
Hybrid cells	BPQD	PTX	Breast cancer	The ability to induce ICD and activate dendritic cells [152]
	PLGA	DHJS	Osteosarcoma	Promotes lethal iron death in cancer cells [153]
	HZGG	CDDP	Colorectal cancer	Inducing apoptosis [154]
	FCM	Fe (II), CpG, MET	Lymph nodes	Antigen delivery to the APC that triggers CTL toxicity to tumor cells [155]

**Table 3:** Clinical trial stage of BNDS.

type	active components	indications	institution/nation	Phase stage	no.
erythrocyte	Tumor Antigen	Hematologic Malignancy	Westlake Therapeutics	Early Phase 1	NCT05707325
erythrocyte	L asparaginase	Acute Lymphoblastic Leukemia	ERYtech Pharmaceuticals	Phase 2	NCT00723346
Macrophages	/	Cervical cancer	Peihua Lu, Wuxi People's Hospital	Observational	NCT05930301
Macrophages	CAR-macrophages	solid tumor	Carisma Therapeutics Inc	Phase 1	NCT04660929
T cell	cyclophosphamide	Sarcoma	M.D. Anderson Cancer Center	Phase 1	NCT06474676
T cell	Cyclophosphamide	prostate cancer	Memorial Sloan Kettering Cancer Center	Phase 1	NCT01140373
T cell	PM-1503-3M	Rabies	Fred Hutchinson Cancer Center	Phase 1	NCT04410900
DC	/	solid tumor	Guangdong 999 Brain Hospital	Phase 1	NCT02808416
DC	/	Glioblastoma	Guangdong 999 Brain Hospital	Phase 1	NCT02709616
DC	Imiquimod	Brain Tumors	Edward Ziga	Phase 1	NCT01902771
Cancer cell	cyclophosphamide	Tumor	National Cancer Institute	Phase 1	NCT01341496
exosome	receptor binding domain	Diabetes mellitus	CureVac	Phase 3	NCT04860258

Overall, BNDS possesses several key advantages: (1) Enhanced target specificity. This specificity not only increases the concentration of the drug at the tumor site but also minimizes the impact on healthy cells, thereby reducing drug-related side effects. (2) Immune evasion and prolonged circulation. The biomimetic membrane-coated nanoparticles can transmit "don't eat me" signals, effectively extending their circulation time in vivo. (3) Excellent biocompatibility. The design reduces the risk of complement activation within the body, ensuring superior biocompatibility.

Although BNDS have demonstrated significant advantages in tumor immunotherapy, they still face several challenges.

(1) Toxicity concerns remain to be addressed. While BNDS can reduce overall drug toxicity through targeted delivery, the metabolism and excretion of drugs within the body, as well as potential drug interactions, must be carefully considered in clinical applications. Core materials used in BNDS, such as gold, silver, and silica, and functionalized modifications of cell membranes, including cross-linking agents, may pose toxicity risks if accumulated excessively in vivo.
(2) The complexity of the preparation process poses a barrier to widespread application. Key steps in constructing BNDS include the extraction of cell membranes, fusion of nanoparticles with cell membranes, and ensuring high extraction efficiency and purity of the membranes. During these processes, membrane proteins are prone to inactivation, which can be exacerbated by parameters such as ultrasonic power, duration, and temperature. Additionally, the low extraction efficiency of cell membranes and the large quantities required for BNDS preparation necessitate optimization of the extraction process and enhancement of purity. Currently, the primary methods for fusing cell membranes with nanoparticles—ultrasonic and extrusion techniques—are still in their early stages of development and present significant drawbacks, posing challenges for industrial-scale production.

However, with industry promotion, more efficient and standardized BNDS preparation equipment and processes will likely become widely adopted. As clinical trials advance, safer and more effective BNDS formulations are expected to achieve further development. Driven by precision medicine and biomanufacturing advancements, bionic delivery systems are poised to enter a period of rapid clinical translation.

## CRediT authorship contribution statement

**Jiawei Yang:** Writing – review & editing, Writing – original draft. **Xueqi Li:** Writing – review & editing. **Tongyu Li:** Writing – review & editing. **Jin Mei:** Project administration, Conceptualization. **Ying Chen:** Writing – review & editing, Writing – original draft, Conceptualization.

## Declaration of competing interest

The authors declare that they have no known competing financial interests or personal relationships that could have appeared to influence the work reported in this paper.

## Data Availability

Data will be made available on request.
